# Subcellular localisation of the p40^*phox*^ component of NADPH oxidase involves direct interactions between the Phox homology domain and F-actin

**DOI:** 10.1016/j.biocel.2010.07.009

**Published:** 2010-10

**Authors:** Dongmin Shao, Anthony W. Segal, Lodewijk V. Dekker

**Affiliations:** a Department of Medicine, University College London, London WC1E 6JJ, UK; b School of Pharmacy, Centre for Biomolecular Sciences, University of Nottingham, Nottingham NG7 2RD, UK

**Keywords:** DMEM, Dulbecco's modified Eagle's medium, GST, glutathione-S-transferase, MALDI-MS, matrix-assisted laser desorption/ionisation-mass spectrometry, PAGE, polyacrylamide gelelectrophoresis, PI(3)P, phosphatidylinositol-3-phosphate, PCR, polymerase chain reaction, PX domain, phox homology domain, SDS, sodium dodecyl sulphate, Cytoskeleton, Neutrophils, Protein interaction, Phosphoinositide, Cell biology

## Abstract

Cytosolic components of the NADPH oxidase interact with the actin cytoskeleton. These interactions are thought to be important for the activation of this enzyme system but they are poorly characterised at the molecular level. Here we have explored the interaction between the actin cytoskeleton and p40^*phox*^, one of the cytosolic components of NADPH oxidase. Full length p40^*phox*^ expressed in COS cells co-localised with F-actin in a peripheral lamellar compartment. The co-localisation was lost after deletion of the Phox homology (PX) domain and the PX domain in isolation (p40PX) showed the same F-actin co-localisation as the full length protein. PX domains are known lipid-binding modules however, a mutant p40PX which did not bind lipids still co-localised with F-actin suggesting that lipid-independent interactions underlie the localisation. Affinity chromatography identified actin as a binding partner for p40PX in neutrophil extracts. Pure actin interacted with both p40^*phox*^ and with p40PX suggesting it is a direct interaction. Disruption of the actin cytoskeleton with cytochalasin D resulted in actin rearrangement and concomitantly the localisation of full length p40^*phox*^ proteins and that of p40PX changed. Thus p40PX is a dual F-actin/lipid-binding module and F-actin interactions with the PX domain dictate at least in part the intracellular localisation of the cytosolic p40^*phox*^ subunit of the NADPH oxidase.

## 1 Introduction

Polymorphonuclear leukocytes (‘neutrophils’) form a major component of the white blood cell mass and have a pivotal role in the innate defence of the body against invading micro-organisms. They execute their function by virtue of ingestion of the microbial species in a process termed phagocytosis and by the concomitant activation of a highly efficient NAPDH oxidase at the invaginated membrane. This NADPH oxidase releases reactive oxygen species into the enclosed vacuolar space. These ultimately participate in the inactivation of the ingested microbe (Reeves et al., 2002; Segal, 2005).

The NADPH oxidase enzyme complex consists of six components: the membrane-bound *phox* proteins (p22^*phox*^ and gp91^*phox*^), the cytosolic *phox* proteins (p40^*phox*^, p47^*phox*^, and p67^*phox*^) and rac GTPase (Segal, 2005). Interactions between these components and with other proteins and lipid factors govern the activation state of the oxidase. The membrane-bound components essentially constitute a transmembrane electron transport chain that transfers electrons from cytosolic NADPH onto vacuolar oxygen thus generating vacuolar oxygen radicals. p67^*phox*^ acts to initiate electron transport (Nisimoto et al., 1999) and it becomes membrane bound upon activation and translocation of p21^*rac*^ which in its membrane-bound form represents a docking site for p67^*phox*^ (Diekmann et al., 1994; Lapouge et al., 2000). p67^*phox*^ and p40^*phox*^ can be isolated as a complex from resting neutrophils by gel filtration (Brown et al., 2003). Under these conditions, p47^*phox*^ appears to be excluded from the larger complex of cytosolic *phox* proteins, even though recombinant p47^*phox*^ readily forms a ternary complex with p40^*phox*^ and p67^*phox*^ in the test tube (Lapouge et al., 2002; Wientjes et al., 1993, 1996). It has been proposed that basal phosphorylation of p47^*phox*^ in resting neutrophils precludes formation of the larger complex and that dephosphorylation of p47^*phox*^ is required for this interaction to take place (Groemping et al., 2003; Groemping and Rittinger, 2005). Further (re)phosphorylation of p47^*phox*^ results in conformational changes and allows (inter)molecular interactions to take place with the p40^*phox*^/p67^*phox*^ complex and with the membrane-bound p22^*phox*^ to assemble an active NADPH oxidase (de Mendez et al., 1997; Kami et al., 2002; Karathanassis et al., 2002; Sumimoto et al., 1996).

Membrane stabilisation and indeed activation of the oxidase also involve phospholipid binding to the Phox homology (PX) domains of p47^*phox*^ and p40^*phox*^ (termed ‘p40PX’ and ‘p47PX’ hereafter) (Bravo et al., 2001; Ellson et al., 2001b; Kanai et al., 2001; Karathanassis et al., 2002; Kuribayashi et al., 2002; Suh et al., 2006). These two PX domains display different lipid specificities so that p40PX recognises phosphatidylinositol-3-phosphate (PI(3)P) and p47PX recognises phosphatidylinositol-3,4-bisphosphate (Kanai et al., 2001). Lipid binding is essential for NADPH oxidase activation *in vitro* and *in vivo*, as shown by mutagenesis of the lipid-binding pocket of p40PX (Ellson et al., 2006a). The PX domains of both proteins are normally occluded (Honbou et al., 2007; Karathanassis et al., 2002; Ueyama et al., 2008). In p47^*phox*^ an interaction with the SH3 and autoinhibitory domains and with residues 341–360 occurs, which is released by phosphorylation of p47^*phox*^ (Karathanassis et al., 2002; Ueyama et al., 2008). In p40^*phox*^, the PB1 domain interacts with p40PX but the exact mechanism by which this is alleviated is as yet unclear (Honbou et al., 2007). p47^*phox*^ is recruited at early stages of phagosome formation and p40^*phox*^ at later stages, possibly as a consequence of availability of different types of phosphoinositides (Condliffe et al., 2005; Ueyama et al., 2007, 2008). This suggests the two proteins may have different functions in the process of NADPH oxidase activation.

In humans, mutations in NADPH oxidase components are associated with specific immune deficiencies (Segal, 2005). No mutants in p40^*phox*^ are known which has made it difficult to appreciate the importance of this protein for oxidase activity. Genetic deletion of p40^*phox*^ results in embryonic lethality suggesting an important developmental role for this protein (Ellson et al., 2006b). Furthermore, neutrophils from heterozygous animals show reduced NADPH oxidase activation compatible with an important function for p40^*phox*^ in NADPH oxidase activation (Ellson et al., 2006b). Interestingly, mutating the lipid-binding site in PX domain of p40^*phox*^ does not have the same effect on NADPH oxidase activity *in vivo* as removing full length p40^*phox*^ suggesting that not all contribution of p40^*phox*^ to NAPDH oxidase activation is via lipid binding to it PX domain (Ellson et al., 2006a,b). It is not clear whether the embryonic function of p40^*phox*^ involves its lipid binding or lipid-independent properties.

Bissonnette et al. (2008) proposed a two-step membrane interaction/activation model for p40^*phox*^, in which a lipid-independent membrane binding event ‘primes’ the oxidase, followed by PI(3)P-induced molecular change and activation of the NADPH oxidase. It was proposed that the membrane binding involved cytoskeletal interactions, although the exact nature of these and the molecular regions involved remained to be identified (Bissonnette et al., 2008). In support of this idea, actin filaments were shown to be required for complete translocation of cytosolic *phox* proteins to the membrane (Allen et al., 1999; Dusi et al., 1996). Furthermore, p40^*phox*^ can be extracted in the Triton-insoluble fraction of resting neutrophils, suggesting a constitutive association with the actin cytoskeleton (El Benna et al., 1999). Recently, Chen et al. (2007) observed that p40^*phox*^ co-immunoprecipitated with actin in extracts from COS-phox cells and suggested that these interactions with F-actin serve to negatively regulate p40^*phox*^. The binding site in p40^*phox*^ responsible for the actin association is not clearly defined. Actin binding proteins moesin and coronin have been shown to bind p40^*phox*^ (Grogan et al., 1997; Wientjes et al., 2001). In the case of moesin the interaction takes place at multiple sites in p40^*phox*^ including the PX domain and the SH3 domain (Wientjes et al., 2001).

Here we explored the relationship between the actin cytoskeleton and p40^*phox*^. We report that the PX domains of the cytosolic *phox* proteins represent a phosphoinositide-independent F-actin binding site. These interactions may serve as anchoring point for NADPH oxidase components in conjunction with phosphoinositide binding to allow accurate spatial control of NADPH oxidase activity during phagocytosis.

## 2 Materials and methods

### 2.1 DNA constructs

DNA fragments representing p40^*phox*^ (PX domain, residues 1–154), p47^*phox*^ (PX domain, residues 1–144), and p67^*phox*^ (residues 300–460) were inserted into the bacterial expression vector pQE9 in frame with an N-terminal his tag. Glutathione-S-transferase (GST)-PX domain constructs were as described (Wientjes et al., 2001). DNA fragments representing full length p40^*phox*^ and p47^*phox*^, p40PX (residues 1–154), p47PX (residues 1–144), p40^*phox*^-ΔPX (residues 155–339), p47^*phox*^-ΔPX (residues 145–390), p40^*phox*^(R58Q), p40PX(R58Q), p47^*phox*^(R43Q), and p47PX(R43Q) were inserted into the mammalian expression vector pEF-LINK in frame with an N-terminal myc-tag.

### 2.2 Cellular expression studies

COS7 cells were maintained in Dulbecco's modified Eagle's medium (DMEM) (GIBCO BRL^®^)/10% Foetal Calf Serum (GIBCO BRL^®^) in 100 μg/ml penicillin and 100 μg/ml of streptomycin (GIBCO BRL^®^) at 37 °C/5% CO_2_. 1 day before transfection, cells were resuspended in DMEM at 1 × 10^5^/ml, seeded on MatTek dishes (MatTek Co.) and incubated at 37 °C/5% CO_2_. For serum starvation, medium was replaced with DMEM/1% Bovine Serum Albumin (Sigma) for 24 h.

Cells were transfected using Lipofectamine-2000 (Invitrogen) with 1–2 μg DNA for 5–6 h. Transfection mixtures were removed and cells were incubated for 18 h in DMEM/10% Foetal Calf Serum and fixed for 5 min at 37 °C with 3.7% formaldehyde in buffer A (5 mM KCl, 138 mM NaCl, 4 mM NaHCO_3_, 0.4 mM KH_2_PO_4_, 1.1 mM Na_2_HPO_4_, 2 mM MgCl_2_, 5 mM PIPES, 2 mM EGTA, 5.5 mM glucose, and pH 6.0–6.1). Cells were permeabilised for 20 min at 20 °C with 0.5% Triton X-100/buffer A, and blocked with 0.1 M glycine/buffer A for 10 min. Cells were washed with buffer B (20 mM Tris–Cl, 150 mM NaCl, and pH 7.5) and incubated with buffer B/1% Bovine Serum Albumin, 1% Foetal Calf Serum and 0.5 μM rhodamine-labelled phalloidin (Molecular Probes) for 30 min. Cells were incubated with 9E10 antibody (Santa Cruz) at 20 °C for 1 h and washed 5 times with buffer B/1% Bovine Serum Albumin. Subsequently, cells were incubated with fluorescein isothiocyanate-labelled donkey anti-mouse (Jackson Immunoresearch laboratories Inc.) at 20 °C for 1 h, washed 5 times with buffer B and mounted in buffer B/50% glycerol and 6 g/l n-propyl gallate. Images were taken on a Bio-Rad confocal microscope. Experiments were repeated 3 times and representative images were taken. Images shown were observed in greater than 80% of the cells inspected over three experiments. For immunoprecipitation, cells from 10 cm dishes were collected 18 h after transfection, lysed in 1 ml of buffer C (50 mM Tris–Cl pH 8, 150 mM NaCl, 1 mM EDTA, 10% glycerol, 1% NP-40, 10 μg/ml leupeptin, 10 μg/ml TLCK, 10 μg/ml aprotinin, 1 mM DFP, and 0.2 mM PMSF) for 30 min on ice, then centrifuged at 4 °C, 13,000 rpm for 30 min, after which the supernatant was pre-incubated with protein A beads for 30 min. Cleaned supernatant was then incubated with 2 μg of anti-myc antibody and 20 μl of protein A beads at 20 °C for 2 h or 4 °C overnight. Beads were washed 5 times with buffer C and boiled in 30 μl of 2× sodium dodecyl sulphate (SDS) sample buffer. 15 μl of each sample was analysed by 10% SDS-polyacrylamide gelelectrophoresis (PAGE) and western blot analysis using an actin antibody (Santa Cruz).

### 2.3 Preparation of proteins

*Escherichia coli* BL21 (Novagen) was co-transfected with pREP4 (Qiagen) and pQE9-p40PX, pQE9-p47PX or pQE9-p67(302–460) respectively, and grown overnight at 37 °C in 100 ml Luria Bertani media containing 100 μg/ml ampicillin and 25 μg/ml kanamycin. Overnight cultures were diluted to 4 l of defined media containing 3.5 g/l K_2_HPO_4_, 5 g/l KH_2_PO_4_, 3.5 g/l (NH_4_)_2_HPO_4_, 10 g/l yeast extract, 10 g/l glucose, 0.5 g/l MgCl_2_·6H_2_O, 100 μg/ml ampicillin and 25 μg/ml kanamycin. The culture was grown in a fermentation tank at 37 °C with an air-flow of 3 l/min and stirring of 300 rpm until OD_600_ = 1–2 (2–3 h) followed by 4–5 h in medium containing 0.25 mM 1 M isopropyl-β-d-thiogalactopyranoside (CALBIOCHEM^®^) after which cells were harvested by centrifugation. Cells were resuspended in 50 ml of buffer D (20 mM HEPES, 300 mM NaCl, pH 7.0)/30 mM imidazole, 1 mg/ml lysozyme, incubated at 4 °C for 45 min after which they were homogenised in a Dounce homogeniser and centrifuged at 28,500 × *g* (Sorvall, SS-34 rotor) at 4 °C for 30 min. The supernatant was incubated with 4–6 ml of 50% slurry Ni^2+^–agarose beads (Novagen) in buffer D/30 mM Imidazole at 4 °C for 30 min. Beads were collected, washed twice with 5 bead volumes of buffer D and proteins were eluted with 4 bead volumes of buffer D/300 mM Imidazole. Eluates were dialysed against buffer E (20 mM HEPES, 100 mM NaCl, pH 7.0) overnight and fractionated by SP-Sepharose 4B^®^ (Pharmacia) fast flow chromatography with a linear gradient of NaCl (0.1–1 M) in buffer E.

The bacterial expression of GST fusion proteins was similar to that of his-tagged proteins. Ten millilitres of BL21 cells were resuspended in 50 ml of buffer F (137 mM NaCl, 2.7 mM KCl, 100 mM Na_2_HPO_4_, 2 mM KH_2_PO_4_, and pH 7.2)/1 mg/ml lysozyme, and a lysate was obtained as above. This was incubated with a 50% slurry of Glutathione-Sepharose 4B^®^ (Pharmacia) at 4 °C for 30 min in buffer F. Beads were collected by centrifugation, washed twice with buffer F and once with buffer E and proteins were eluted with 4 bed volumes of buffer E/10 mM reduced glutathione. Proteins were purified by SP-Sepharose 4B^®^ or Q-Sepharose 4B^®^ fast flow chromatography with a 0.1–1 M NaCl gradient in buffer E.

### 2.4 Preparation of neutrophil cytosol and identification of p40PX binding proteins

Neutrophils were prepared from buffy coat residues or buffy coats (North London Blood Transfusion Service) as described (Davidson-Moncada et al., 2002). Cells were resuspended in 4 volumes of buffer G (10 mM HEPES, 5 mM NaCl, 10 mM KCl, 5 mM MgCl_2_, pH 7.0) and lysed using a Nitrogen Cavitation Bomb at 50 bar for 30 min at 4 °C followed by centrifugation at 400 × *g* for 10 min. The post-nuclear supernatant was centrifuged at 417,000 × *g* for 30 min at 4 °C. The supernatant (cytosol) was used immediately or stored at −70 °C.

Neutrophil cytosol (10 mg/ml protein) was incubated with Glutathione-Sepharose 4B^®^ for 30 min at 4 °C and the beads removed by centrifugation at 1000 × *g* for 5 min. This pre-absorbed cytosol (1 ml) was incubated either with 0.1 ml of GST-P40PX-coupled Glutathione-Sepharose 4B^®^ or 0.1 ml of GST-Glutathione-Sepharose 4B^®^ for 1 h at room temperature. Beads were washed with buffer G, resuspended in 2× SDS sample buffer and samples were analysed by 10% SDS-PAGE. To identify proteins of interest, gel bands were excised, destained with 40% ethanol, subjected to in-gel trypsin digestion and matrix-assisted laser desorption/ionisation-mass spectrometry (MALDI-MS) as described (Paclet et al., 2004).

### 2.5 F-actin binding assay

Non-muscle actin (>99% pure) was purchased from Cytoskeleton Ltd. G-actin was cleared at 100,000 × *g* for 30 min and polymerised at a concentration of 20 μM at 20 °C for 1 h in buffer H (20 mM HEPES, 100 mM NaCl, 1 mM MgCl_2_, 0.5 mM ATP, 0.5 mM DTT, pH 7.0). PX domains were dialysed against buffer H overnight at 4 °C and centrifuged at 100,000 × *g* at 20 °C for 30 min (Beckmann, TLA100.2 rotor). 50 μl of supernatants (500 pmol) were then mixed with an equal volume of F-actin (100 pmol), incubated at 20 °C for 1 h and centrifuged at 100,000 × *g* for 30 min at 20 °C. The supernatants were removed carefully and the pellets were rinsed with 100 μl buffer H and resuspended in 100 μl buffer H. Equal volumes of supernatants and resuspended pellets were analysed by SDS-PAGE.

## 3 Results and discussion

### 3.1 p40PX mediates co-localisation of p40^phox^ with F-actin rich lamellipodia in intact cells

In order to understand the interaction between p40^*phox*^ and the actin cytoskeleton, mammalian expression constructs were generated and localisation studies were performed in COS cells. A myc-tag was engineered onto the p40^*phox*^ coding domains. Cells were seeded on cover slips 24 h before transfection, and then cultured for 18 h after transfection in serum-containing medium, favouring a well established leading edge.

**Fig. 1:**
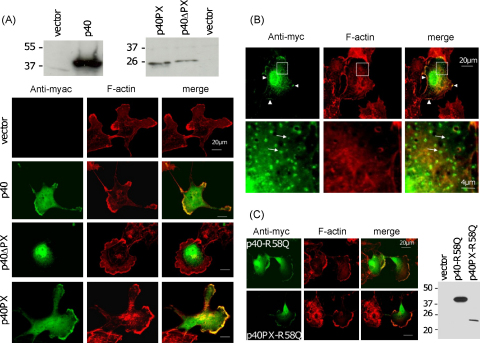
PX domains direct p40^*phox*^ to F-actin rich lamellipodia independent of lipid binding. (A) COS7 cells were transfected with empty pEF-myc vector, pEF-myc-p40^*phox*^ (‘p40’), pEF-myc-p40PX (‘p40PX’) or pEF-myc-p40ΔPX (‘p40ΔPX’). Top panels: cells were extracted in SDS buffer and analysed by SDS-PAGE and western blotting using the 9E10 myc monoclonal antibody. The position and molecular mass (kDa) of marker proteins is indicated. Lower panels: cells were stained with the 9E10 myc monoclonal antibody (left column) or rhodamine-phalloidin (middle column). The right column shows the overlap in fluorescence signal (green: myc, red: actin, yellow: co-localisation). A representative cell is shown for each transfection. Scale bars: 20 μm. (B) COS7 cells were transfected with pEF-p40PX and 18 h after transfection cells were starved for 24 h and stained with the 9E10 myc monoclonal antibody (left column) or rhodamine-phalloidin (middle column) to visualize myc-tagged PX domain and actin filaments respectively. The right column shows the overlap in fluorescence signal (green: myc; red: actin; yellow: co-localisation). The bottom row shows magnifications of the areas outlined in the top row. Arrows indicate perinuclear vesicle and arrowhead indicates lamellar staining. (C) Left panel: COS7 cells were transfected with pEF-myc-p40^*phox*^-R58Q (‘p40-R58Q’) or pEF-myc-p40PX-R58Q (‘p40PX-R58Q’) and stained with the 9E10 myc monoclonal antibody (left column) or rhodamine-phalloidin (middle column). The right column shows the overlap in fluorescence signal (green: myc; red: actin; yellow: co-localisation). A representative cell is shown for each transfection. Right panel: cells were extracted in SDS buffer and analysed by SDS-PAGE and western blotting using the 9E10 myc monoclonal antibody. The position and molecular mass (kDa) of marker proteins is indicated. Scale bars: 20 μm.

Western blots confirmed expression of myc-tagged p40^*phox*^ in COS cells (Fig. 1A). Confocal immunofluorescence microscopy revealed that p40^*phox*^ localised to a peripheral actin rich lamellar compartment in the cells (Fig. 1A). Deletion of the PX domain abolished the localisation pattern, with the expressed deletion mutant now residing in the nucleus (Fig. 1A). This indicates that the PX domain dictates the subcellular localisation of p40^*phox*^ and is a determinant in co-localisation of this protein with the F-actin cytoskeleton inside the cell. The nuclear localisation may be driven by unmasking of multiple basic residues after deletion of the PX domain.

The isolated myc-tagged p40^*phox*^-PX domain (‘p40PX’) expressed well in COS cells and was found to co-localise with F-actin (Fig. 1A), which was predominantly present at the cellular lamellae. The pattern of localisation of the myc-tagged PX domain completely matched that of the full length proteins (Fig. 1A). Thus p40^*phox*^ can co-localise with the actin cytoskeleton in COS cells and the PX domain in p40^*phox*^ is a major determinant in this localisation pattern. The localisation pattern is consistent with observations that p40^*phox*^ co-immunoprecipitates with actin in COS-phox cells (Chen et al., 2007) and that it fractionates in the (actin rich) Triton-insoluble fraction in neutrophils (El Benna et al., 1999). Furthermore the localisation pattern is reminiscent of that observed for GFP-p40^*phox*^ after transfection into COS-phox cells (Chen et al., 2007). However, the latter is dependent on p67^*phox*^, which was not present in our current cell system. Thus either the localisation pattern observed in COS-phox cells is in fact different from the present pattern, or subcortical actin co-localisation can in principle occur in the absence of p67^*phox*^ and depends for instance on the morphological features of the cells.

### 3.2 The interaction between p40PX and F-actin rich lamellipodia does not require lipids

Observations in the literature indicate that the GFP-tagged p40PX domain localises to PI(3)P-rich intracellular structures (Bravo et al., 2001; Ellson et al., 2001a; Stahelin et al., 2003; Zhan et al., 2002). Since these studies were generally done in serum-starved cells, we investigated the effect of serum starvation on the localisation of p40PX in our system. Under these conditions additional perinuclear vesicular localisation of the isolated p40PX domain was observed. The pattern of vesicle staining is highly reminiscent of that observed in COS cells after expression of a dominant negative form of PIKfyve, which normally converts PI(3)P into PI(3,5)P_2_ (Ikonomov et al., 2006). Thus the intracellular vesicles may represent a pool of PI(3)P-positive perinuclear endosomes. These then recruit myc-tagged p40PX in agreement with its known PI(3)P-binding properties. The titration of p40PX into these vesicles affects the amount of protein available to associate with lamellar F-actin, however significant co-localisation of p40PX with this actin pool was still observed (Fig. 1B). Thus this experiment indicates that in our system co-localisation of p40PX with both PI(3)P and F-actin can be observed. The fact that in contrast to the vesicle localisation of p40PX, its co-localisation with F-actin was observed regardless of serum conditions (Fig. 1) suggests that PI(3)P lipids may not be required for the latter.

To define the lipid requirement for F-actin binding more explicitly we employed the R58Q mutant, known to be deficient in PI(3)P and endosome binding (Bravo et al., 2001). We generated R58Q mutants of full length p40^*phox*^ protein and of the isolated p40PX domain. Taking into account relative expression levels of these mutant proteins, their distribution patterns were not different from those of the non-mutated proteins (Fig. 1A and C) and co-localisation with lamellar F-actin was still observed. Thus lipid binding is not involved in the co-localisation of PX domains with F-actin and direct, lipid-independent protein interactions may dictate the localisation of *phox* proteins and PX domains to F-actin rich areas.

### 3.3 Identification of actin as PX binding partner

**Fig. 2:**
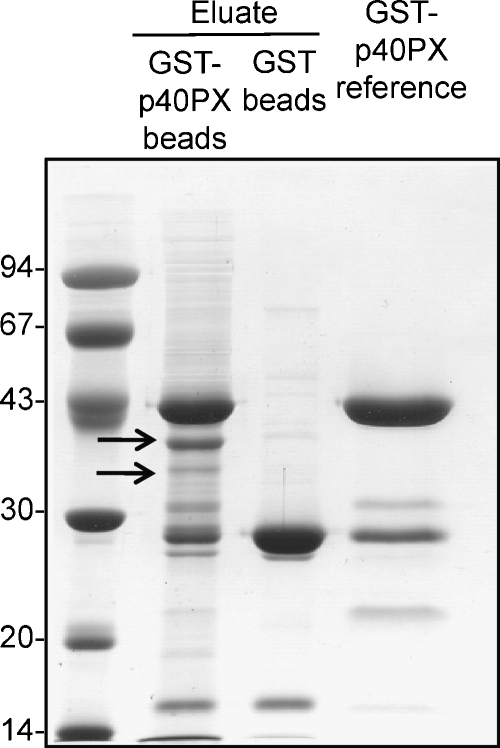
Identification of actin as PX binding partner. Neutrophil cytosol was pre-absorbed with Glutathione-Sepharose^®^ 4B beads and then incubated with GST-p40PX-Glutathione-Sepharose^®^ 4B beads or Glutathione-Sepharose^®^ 4B beads as described in Section 2. Bound proteins were recovered and resolved by SDS-PAGE. The gel was stained with Coomassie blue R-250. Proteins marked by arrows were cut from the gel and digested in-gel by trypsin at 30 °C for 12 h. The first lane shows markers proteins with the molecular mass (kDa) indicated. The fourth lane shows the preparation of GST-p40PX used to immobilise.

Several actin binding proteins have been identified as partners for p40^*phox*^. These include the protein moesin. Moesin interaction takes place at the PX domain, but also at the isolated SH3 domain (Wientjes et al., 2001). Since the p40^*phox*^-ΔPX mutant (which still retains an SH3 domain), did not co-localise with F-actin in the cell (Fig. 1A), it appears that moesin is not the partner that dictates this localisation and we concluded that other protein interactions may be important for F-actin targeting. To identify such partners for p40PX, affinity chromatography experiments were performed. Neutrophils were employed as cellular source since these cells provide the physiological context of the *phox* proteins and it was reasoned that they represented the most logical source of potential non-lipid-binding partners. Neutrophil extracts were incubated with GST or GST-p40PX affinity matrices and bound proteins were analysed by SDS-PAGE (Fig. 2). Two gel bands were retained on the GST-PX column but not on the GST column. These proteins were cut from the gel and subjected to in-gel trypsin digestion and MALDI-MS. This analysis identified both proteins as β-actin (Table 1, supplement) with the smaller protein most likely representing a breakdown product generated during extraction. The immobilisation of actin on the PX domain matrix observed here indicates that actin itself may serve as a direct binding partner for the PX domain.

### 3.4 F-actin interacts directly with PX domain of p40^*phox*^

**Fig. 3:**
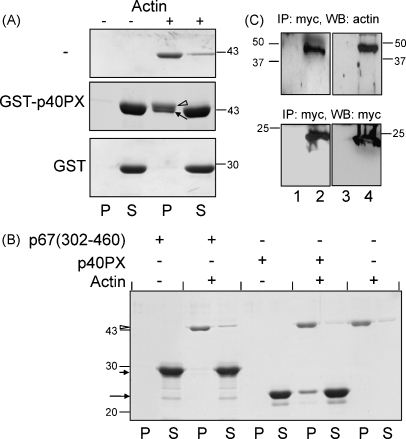
PX domains bind F-actin directly and independent of lipid binding. (A) 50 μM of GST-p40PX, 50 μM GST or buffer H were incubated with or without 10 μM of F-actin in a final volume of 100 μl and left at room temperature for 30 min. The mixtures were centrifuged at 100,000 × *g* for 30 min and the pellets were resuspended in 100 μl buffer H. Ten microlitres of pellet (‘P’) or supernatant (‘S’) were analysed by SDS-PAGE and stained with Coomassie blue R-250. Open arrowhead indicates actin; arrow indicates GST-p40PX. The position and molecular mass (kDa) of marker proteins is indicated. (B) 50 μM of his-p40PX, 50 μM his-p67(300–460) or buffer H were incubated with or without 10 μM of F-actin in a final volume of 100 μl and incubated at room temperature for 30 min. The mixtures were centrifuged at 100,000 × *g* for 30 min and the pellets were resuspended in 100 μl buffer H. Ten microlitres of pellet (‘P’) or supernatant (‘S’) were analysed by SDS-PAGE and stained with Coomassie blue R-250. The position and molecular mass (kDa) of marker proteins is indicated. Open arrowhead indicates actin; short arrow indicates his-p67(300–460) long arrow indicates his-p40PX. (C) COS7 cells were transfected with pEF (lanes 1 and 3), pEF-p40PX (lane 2) or pEF-p40PX-R58Q (lane 4) and lysed 24 h after transfection. Immunopreciptation was then carried out using an anti-myc antibody followed by SDS-PAGE and western blotting with anti-actin and anti-myc as indicated. The position and molecular mass (kDa) of marker proteins is indicated.

Since actin was the predominant immobilised protein on the GST-affinity matrix (no other immobilised proteins were observed at similar levels) it is likely that actin interacts directly with PX domains. However, the data do not completely exclude indirect interactions. To investigate this, binding assays were performed using pure (>99%) non-muscle actin. The experiment was performed in buffer conditions that allow actin to polymerise into the F-actin form and co-sedimentation of PX with this F-actin at high centrifugation speed was used as readout for actin binding. This method has the advantage that it monitors F-actin binding in a native F-actin conformation. In the absence of added protein, actin was largely present in the pellet, indicating that actin polymerisation occurred under these conditions (Fig. 3A). There was a significant shift of GST-p40PX from the supernatant to the pellet in the presence of F-actin, whilst no such shift was found for the GST tag itself (Fig. 3A). Thus the GST fusion tag itself does not co-sediment with F-actin but GST-PX fusion proteins do. In order to exclude effects of the tag, the experiment was repeated with the same PX domains fused to a 6×histidine tag was chosen. His-tagged fusion proteins were purified and their molecular weights were confirmed to be close to their predicted molecular weights using electrospray mass spectrometry (not shown). Co-sedimentation of his-p40PX with F-actin was observed (Fig. 3B) but this was not the case for his-p67(302–460) which lacks a PX domain. Thus the isolated p40PX domain binds to pure F-actin. Binding of actin to these domains was observed in the absence of any additional proteins or lipid factors and it can therefore be concluded that PX domains interact directly with F-actin.

To prove such interaction occurred at the cellular level, we transfected COS cells with p40PX, and its non-lipid-binding mutants and retrieved the proteins by immunoprecipitation. In all cases, actin co-immunoprecipitated with myc-p40PX and its R58Q mutant (Fig. 3C), suggesting that cellular F-actin binding is independent of PI(3)P binding. Previous studies indicated that GST-PX does not bind G-actin (unassembled monomeric actin) (Zhan et al., 2004). Since these studies employed G-actin and the current studies employed F-actin, it appears that the actin conformation is critical for its ability to interact with p40PX.

### 3.5 p40^*phox*^ protein and p40PX localisation is governed by F-actin cytoskeleton

**Fig. 4:**
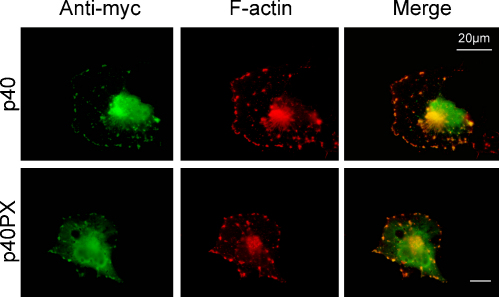
Disruption of F-actin leads to relocalisation of full length p40^*phox*^ and p40PX. COS7 cells were transfected with pEF-myc-p40^*phox*^ (‘p40’) or pEF-p40PX (‘p40PX’) and incubated at 37 °C for 16 h. Cells were then treated with 10 μM cytochalasin D for 20 min after which they were fixed and stained with the 9E10 myc monoclonal antibody (green signal) or rhodamine-phalloidin (red signal). Orange/yellow signal indicates overlap between the myc-tag and F-actin. A representative cell is shown for each transfection. Scale bars: 20 μm.

Our data suggest that F-actin may serve as intracellular binding site for p40^*phox*^. This is consistent with the two-step model by Bissonnette et al in which p40^*phox*^ activates the NADPH oxidase via cytoskeletal binding and subsequent PI(3)P lipid binding (Bissonnette et al., 2008). To further establish that F-actin acts as recognition site for p40PX, the F-actin cytoskeleton was disrupted with cytochalasin D and the localisation of p40^*phox*^ was determined. Treatment with cytochalasin D resulted in the loss of the strong lamellipodial F-actin signal as observed in control cells (Fig. 1) with F-actin becoming confined to smaller membrane patches (Fig. 4). myc-p40^*phox*^ as well as myc-p40PX underwent the same subcellular rearrangement as F-actin, co-localising with these membrane patches (Fig. 4). Thus even after allowing for the fact that our cytochalasin treatment only partially affected F-actin, it is clear that F-actin is a determinant in the localisation of p40PX and p40^*phox*^. An interaction with F-actin could explain the cytoskeletal priming interaction of p40^*phox*^ in activation of NADPH oxidase as cited above.

### 3.6 p47PX has a similar lipid-independent F-actin binding function

**Fig. 5:**
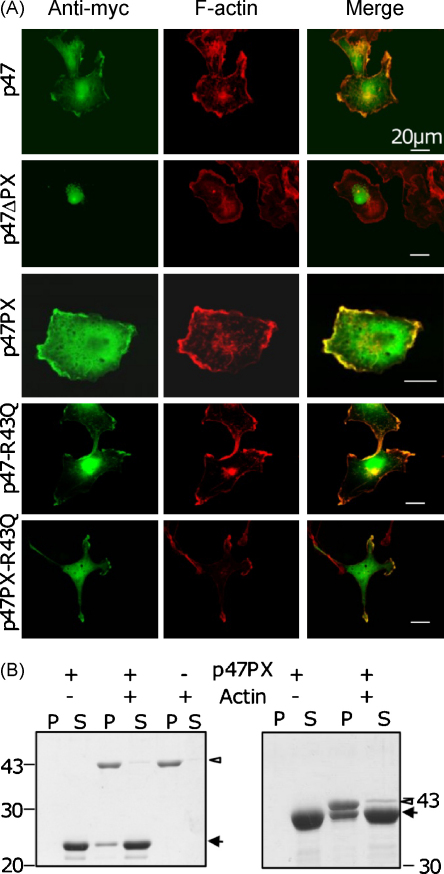
p47^*phox*^ and p47PX display lipid-independent F-actin interacting properties. (A) COS7 cells were transfected with pEF-myc-p47^*phox*^ (‘p47’), pEF-myc-p47^*phox*^-ΔPX (‘p47ΔPX’), pEF-p47PX (‘p47PX’), pEF-myc-p47^*phox*^-R43Q (‘p47-R43Q’) or pEF-myc-p47PX-R43Q (‘p47PX-R43Q’) and incubated at 37 °C for 16 h. Cells were then fixed and stained with the 9E10 myc monoclonal antibody (green; left column) or rhodamine-phalloidin (red; middle column). Orange/yellow signal indicates overlap between the myc-tag and F-actin (right column). A representative cell is shown for each transfection. Scale bars: 20 μm. (B) 50 μM of his-p47PX (left panel) or GST-p47PX (right panel) were mixed with 10 μM of F-actin (+) or buffer H (−) in a final volume of 100 μl and left at room temperature for 30 min. Pellets (‘P’) and supernatants (‘S’) were then obtained and analysed as described in Section 2. Open arrowheads: actin; filled arrows: p47PX fusion protein. The position and molecular mass (kDa) of marker proteins is indicated.

Recent studies suggested that the lipid-independent function of p40PX could be replaced by a very similar function of p47PX (Bissonnette et al., 2008). If, given the observations above, F-actin binding represented this lipid-independent function of p40PX, it would be predicted that the p47PX domain is also a lipid-independent actin anchoring point. To test this we generated myc-tagged expression constructs for p47^*phox*^. Similar to the behaviour of p40^*phox*^, p47^*phox*^ localises to F-actin rich lamellae in COS cells (Fig. 5A). Deletion of the PX domain results in a loss of F-actin localisation whilst p47PX itself in isolation associates with F-actin rich lamellipodia. Importantly, the R43Q mutant of p47^*phox*^, which is deficient in lipid binding, localises to F-actin rich membrane (Fig. 5A). Furthermore, both his- and GST-tagged p47PX co-sedimented with F-actin filaments (Fig. 5B). Thus lipid-independent F-actin binding properties are displayed by PX domains from both *phox* proteins. This may explain why a p47PX-p40^*phox*^ chimaeric fusion protein can rescue NADPH oxidase activity in neutrophil cores which had been p40^*phox*^-depleted (Bissonnette et al., 2008).

Thus far, PX domains of the cytosolic *phox* proteins have primarily been thought of as phosphoinositide binding sites. We showed that these domains also bind F-actin and this needs to be considered when interpreting their localisation and function. More generally, protein interaction functions for PX domains have been established using yeast two hybrid approaches (Vollert and Uetz, 2004). Various studies suggest that the lipid-binding pocket of the PX domain of p40^*phox*^ is occluded by intramolecular interactions (Honbou et al., 2007; Karathanassis et al., 2002). F-actin binding observed here appears to occur at a different surface of the PX domain since (i) the p40PX-R58Q mutant retains F-actin binding properties, suggesting that the lipid-binding part of the domain is not involved in F-actin binding and (ii) the F-actin binding surface appears not occluded, since p40^*phox*^ localises to F-actin after transfection. Thus the PX domain has at least two interaction surfaces, one for phospholipids and a second for F-actin.

Our current observations suggest that if areas of high F-actin polymerisation are present in the cell, p40^*phox*^ and p47^*phox*^ will become targeted to these areas via direct interactions of the PX domain and F-actin. Using immunofluorescence we observed extensive F-actin accumulation at the site of microbial intake in neutrophils (Lopez-Lluch et al., 2001). F-actin accumulates at the nascent phagosome and is subsequently shed upon its maturation (Allen et al., 1999). F-actin at the phagosomal membrane could thus act as initial signal for binding of p47^*phox*^ and p40^*phox*^, via their PX domains. Indeed, p47^*phox*^ is recruited to nascent phagosomes in an F-actin dependent manner (Allen et al., 1999). PKC-β and PKC-δ are recruited to the phagosome and are essential for activation of neutrophil NADPH oxidase most notably by phosphorylating p47^*phox*^ (Chou et al., 2004; Dekker et al., 2000; Lopez-Lluch et al., 2001). F-actin binding of p47^*phox*^ may serve to juxtapose it to phagosomal PKC-β and PKC-δ so that it can be phosphorylated. Initial phosphorylation may not lead to membrane stabilisation through p22^*phox*^ binding but on progressive phosphorylation p47^*phox*^ would become fully membrane-stabilised (measurable by fractionation), leading to full NADPH oxidase activation (Rotrosen and Leto, 1990). For p40^*phox*^, F-actin interactions with the PX domain may prime it so that it can bind to PI(3)P generated during phagosome maturation (Bissonnette et al., 2008). In this form, p40^*phox*^ may stabilise the ternary complex at the phagosomal membrane in mature phagosomes (Ueyama et al., 2008). Thus F-actin binding to the cytosolic proteins may increase spatiotemporal control over the activation of NADPH oxidase during phagocytosis.

Intriguingly, genetic deletion of p40^*phox*^ is associated with embryonic lethality (Ellson et al., 2006b) whereas deletion of p47^*phox*^ or the X-linked p91^*phox*^ is not (Jackson et al., 1995). One possibility suggested by the present observations is that cytoskeletal interaction of p40^*phox*^ may have functions during embryogenesis which are separate from the regulation of the phagocyte NADPH oxidase. Further research is needed to establish this idea.
